# Preoperative geriatric nutritional risk index is a useful prognostic indicator in elderly patients with gastric cancer

**DOI:** 10.18632/oncotarget.27635

**Published:** 2020-06-16

**Authors:** Noriyuki Hirahara, Takeshi Matsubara, Yusuke Fujii, Shunsuke Kaji, Ryoji Hyakudomi, Tetsu Yamamoto, Yuki Uchida, Yoshiko Miyazaki, Kazunari Ishitobi, Yasunari Kawabata, Yoshitsugu Tajima

**Affiliations:** ^1^Department of Digestive and General Surgery, Shimane University Faculty of Medicine, Izumo, Shimane, Japan

**Keywords:** geriatric nutritional risk index, overall survival, cancer-specific survival, gastric cancer, elderly patients

## Abstract

Background: The geriatric nutritional risk index (GNRI) was developed to evaluate the prognosis in elderly hospitalized patients at risk of malnutrition and related morbidity and mortality. This study aimed to evaluate the relationship between preoperative GNRI and long-term outcomes in elderly gastric cancer patients.

Materials and Methods: We retrospectively reviewed 297 consecutive patients aged ≥ 65 years who underwent laparoscopic gastrectomy with R0 resection and evaluated their overall survival (OS) and cancer-specific survival (CSS).

Results: In the univariate analyses, OS was significantly associated with the American Society of Anesthesiologists Physical Status (ASA-PS), tumor size, tumor differentiation, pathological stage, carcinoembryonic antigen (CEA), C-reactive protein, postoperative complications, and GNRI, whereas in the univariate analyses of CSS, ASA-PS, tumor size, tumor differentiation, pathological stage, CEA, postoperative adjuvant chemotherapy, and GNRI were significantly associated with poor prognosis. In the multivariate analysis, ASA-PS, tumor differentiation, pathological stage, and GNRI were significant independent prognostic factors of OS, whereas ASA-PS, pathological stage, and CEA were significant independent prognostic factors of CSS.

Conclusions: GNRI is significantly associated with OS and CSS in elderly gastric cancer patients and is an independent predictor of OS. It is a simple, cost-effective, and promising nutritional index for predicting OS in elderly patients.

## INTRODUCTION

The tumor–node–metastasis (TNM) staging has been the global standard for estimating cancer cell dissemination [1]. Nonetheless, TNM classification alone is not sufficient in predicting cancer outcomes because various factors, including tumor characteristics, systemic inflammation, and nutritional status, are involved in tumor progression [2, 3].

The impact of the nutritional status on the outcome of cancer patients has been intensively studied in recent years, and several assessment tools have been proposed for nutritional screening [4]. However, the usefulness of these tools has not been fully evaluated in elderly patients. The geriatric nutritional risk index (GNRI) was developed as a simple and objective nutritional assessment tool for hospitalized elderly patients (aged ≥ 65 years, according to World Health Organization definition of elderly; https://www.who.int/) based on their body weight and serum albumin level [5]. Albumin is affected by age-related changes such as hydration and hepatic and renal dysfunction [6–8]. In addition, body weight appears to be a good indicator of both the severity of systemic illness and the amount of protein-calorie stores [9, 10]. We, therefore, believe that the GNRI accurately reflects the nutritional status of elderly cancer patients who are at risk of malnutrition because of their physiological frailty and vulnerability.

The principal aim of this study was to evaluate the prognostic significance of the preoperative GNRI for estimating the postoperative outcomes of elderly gastric cancer patients.

## RESULTS

### GNRI and clinicopathological features

The relationship between the GNRI values and the clinicopathological characteristics of the 297 patients enrolled in this study is summarized in Table 1. Based on the GNRI cutoff value for overall survival (OS), 97 (32.7%) and 200 (67.3%) patients were classified as having a low or high GNRI, respectively. There was a significant association between the GNRI and clinicopathological factors such as age (*p* < 0.001), body mass index (BMI) (*p* < 0.001), American Society of Anesthesiologists physical status (ASA-PS) (*p* = 0.014), red blood cell (RBC) count (*p* < 0.001), albumin (*p* < 0.001), C-reactive protein (CRP) (*p* < 0.001), tumor size (*p* < 0.001), tumor differentiation (*p* = 0.021), depth of tumor (*p* < 0.001), pathological stage (*p* < 0.001), and intraoperative blood loss (*p* = 0.048).

**Table 1:** Relationships between GNRI and clinicopathological features in all elderly patients with gastric cancer

Characteristics	Number of patients	GNRI (based on OS)			GNRI (based on CSS)		
		< 94.8	≥ 94.8		< 90.9	≥ 90.9	
		(*n* = 97)	(*n* = 200)	*p* value	(*n* = 66)	(*n* = 231)	*p* value
Age (years old)		79 (65–91)	74 (65–89)	< 0.001	79 (65–91)	75 (65–89)	< 0.001
Gender				0.601			0.441
Male	205	65	140		43	162	
Female	92	32	60		23	69	
BMI		20.8 (14.7–30.5)	22.9 (16.5–40.4)	< 0.001	20.1 (14.7–30.5)	22.8 (16.5–40.4)	< 0.001
ASA-PS				0.014			0.069
1	8	0	8		0	8	
2	256	80	176		54	202	
≥ 3	33	17	16		12	21	
WBC (μl)		5450 (1830–13700)	5695 (510–9830)	0.260	5520 (1830–13700)	5680 (510–10300)	0.833
RBC (× 10^4^ μl)		365 (142–570)	435 (203–579)	< 0.001	367 (142–570)	427 (203–579)	< 0.001
Albumin (g/dl)		3.4 (1.1–4.0)	4.2 (3.6–5.0)	< 0.001	3.2 (1.1–3.9)	4.1 (3.4–5.0)	< 0.001
CRP (mg/l)		0.16 (0.01–11.1)	0.07 (0.01–7.09)	< 0.001	0.24 (0.01–11.1)	0.07 (0.01–7.09)	< 0.001
Location of tumor				0.119			0.510
EGJ	11	1	10		1	10	
U	53	22	31		15	38	
M	122	35	87		26	96	
L	111	39	72		24	87	
Tumor size (mm)		58 (7–180)	36 (3–176)	< 0.001	60 (7–170)	40 (3–180)	< 0.001
Procedure				0.118			0.043
LTG	63	27	36		21	42	
LPG	29	7	22		4	25	
L(A)DG	205	63	142		41	164	
Tumor differentiation				0.021			0.101
Well	65	12	53		9	56	
Moderate	120	43	77		26	94	
Poor	112	42	70		31	81	
Depth of tumor				< 0.001			< 0.001
T1a-1b	145	31	114		19	126	
2	44	12	32		7	37	
3	44	24	20		17	27	
4a-4b	64	30	34		23	41	
Lymph node metastasis				0.145			0.158
N0	192	57	135		38	154	
N1	38	11	27		8	30	
N2	35	17	18		13	22	
N3	32	12	20		7	25	
Pathological stage				< 0.001			< 0.001
1a-1b	171	38	133		24	147	
2a-2b	53	26	27		18	35	
3a-3c	73	33	40		24	49	
Operation time (min)		390 (204–911)	409 (70–808)	0.396	392 (204–911)	406 (70–808)	0.643
Intraoperative blood loss		100 (0–1600)	45 (0–4070)	0.048	125 (0–1600)	50 (0–4070)	0.029
CEA (ng/ml)		3.4 (0.8–163.3)	3.4 (0.7–161.1)	0.314	3.7 (0.8–163.3)	3.3 (0.7–161.1)	0.078
Postoperative complications				0.292			0.387
absent	202	62	140		42	160	
present	95	35	60		24	71	
Adjuvant chemotherapy				0.737			0.888
Yes	79	27	52		18	61	
No	218	70	148		48	170	

Abbreviations: OS: overall survival, CSS: cancer-specific survival, GNRI: geriatric nutritional risk index, BMI: body mass index, ASA-PS: American Society of Anesthesiologists physical status, WBS: white blood cell, RBC: red blood cell, CRP: C-reactive protein, EGJ: esophagogastric junction, U: upper part, M: middle part, L: lower part, CEA: carcinoembryonic antigen, LTG: laparoscopic total gastrectomy, LPG: laparoscopic proximal gastrectomy, L(A)Dg: laparoscopic (assisted) distal gastrectomy.

Based on the GNRI cutoff value for cancer-specific survival (CSS), 66 (22.2%) and 231 patients (77.8%) were classified as having a low or high GNRI, respectively. GNRI values were significantly associated with age (*p* < 0.001), BMI (*p* < 0.001), RBC (*p* < 0.001), albumin (*p* < 0.001), CRP (*p* < 0.001), tumor size (*p* < 0.001), operative procedure (*p* = 0.043), depth of tumor (*p* < 0.001), pathological stage (*p* < 0.001), and intraoperative blood loss (*p* = 0.029).

Analysis based on the GNRI cutoff value for OS and CSS showed no causal relationship between GNRI values and postoperative complication rates.

### Cox regression analysis of OS

In univariate analyses, OS was found to be significantly associated with ASA-PS (*p* < 0.001), tumor size (*p* = 0.018), tumor differentiation (*p* < 0.001), pathological stage (*p* < 0.001), carcinoembryonic antigen (CEA) (*p* = 0.005), CRP (*p* = 0.030), postoperative complications (*p* = 0.002), and GNRI (*p* < 0.001). Multivariate analysis revealed that ASA-PS (hazard ratio [HR]: 3.755; 95% confidence interval [CI]: 2.130–6.619; *p* < 0.001), tumor differentiation (HR: 1.798; 95% CI: 1.132–2.857; *p* = 0.013), pathological stage (HR: 2.028; 95% CI: 1.176–3.498; *p* = 0.011), and GNRI (HR: 2.350; 95% CI: 1.436–3.847; *p* < 0.001) were significant independent prognostic factors of OS (Table 2).

**Table 2:** Univariate and multivariate analyses of clinicopathological factors for overall survival

Variables	Category or characteristics	Patients	Univariate analysis			Multivariate analysis		
		(*n* = 297)	HR	95% CI	*p* value	HR	95% CI	*p* value
Gender	(female/male)	92/205	1.158	0.718–1.866	0.547			
ASA-PS	(< 3 / ≥ 3)	264/33	3.937	2.306–6.721	< 0.001	3.755	2.130–6.619	< 0.001
BMI	(≥ 18.5 / < 18.5)	269/28	1.652	0.874–3.122	0.122			
Tumor size	(< 5 / ≥ 5)	163/134	1.706	1.097–2.653	0.018	1.123	0.641–1.970	0.685
Differentiation	(well & mod/poor)	184/113	2.132	1.375–3.305	< 0.001	1.798	1.132–2.857	0.013
Pathological stage	(1,2/3)	224/73	2.720	1.751–4.225	< 0.001	2.028	1.176–3.498	0.011
CEA	(< 5.0 / ≥ 5.0)	225/72	1.936	1.222–3.066	0.005	1.593	0.993–2.557	0.054
CRP	(≦ 0.5 / > 0.5)	248/49	1.750	1.055–2.904	0.030	1.170	0.656–2.088	0.595
GNRI	(> 94.8 / < 94.8)	200/97	2.470	1.596–3.823	< 0.001	2.350	1.436–3.847	< 0.001
Complications	(absent/present)	202/95	1.985	1.275–3.089	0.002	1.532	0.961–2.440	0.073
Intraope. blood loss	(< 50 / ≥ 50)	(163/134)	1.615	0.998 – 2.615	0.051			
Adjuvant Chemo	(no/yes)	218/79	1.544	0.979–2.434	0.062			

Abbreviations: HR: hazard ratio, CI: confidence interval, ASA-PS: American Society of Anesthesiologists physical status, BMI: body mass index, mod: moderately, poor: poorly, CEA: carcinoembryonic antigen, CRP: C-reactive protein, GNRI: geriatric nutritional risk index, Complications: postoperative complications, Intraope. blood loss: intraoperative blood loss, Adjuvant chemo: adjuvant chemotherapy.

### Cox regression analysis of CSS

The univariate analyses identified that CSS was significantly associated with ASA-PS (*p* = 0.014), tumor size (*p* = 0.001), tumor differentiation (*p* = 0.012), pathological stage (*p* < 0.001), CEA (*p* = 0.002), postoperative adjuvant chemotherapy (*p* < 0.001), and GNRI (*p* = 0.006). The ASA-PS (HR: 3.034; 95% CI: 1.272–7.234; *p* = 0.012), pathological stage (HR: 4.178; 95% CI: 1.714–10.183; *p* = 0.002), and CEA (HR: 1.995; 95% CI; 1.016–3.914; *p* = 0.045) were significant independent prognostic factors of CSS in the multivariate analysis. However, the GNRI was not confirmed to be an independent prognostic factor for CSS (Table 3).

**Table 3:** Univariate and multivariate analyses of clinicopathological factors for cancer-specific survival

Variables	Category or characteristics	Patients	Univariate analysis			Multivariate analysis		
		(*n* = 297)	HR	95% CI	*p* value	HR	95% CI	*p* value
Gender	(female/male)	92/205	1.433	0.673–3.048	0.351			
ASA	(< 3 / ≥ 3)	264/33	2.839	1.237–6.519	0.014	3.034	1.272–7.234	0.012
BMI	(≥ 18.5 / < 18.5)	269/28	1.381	0.487–3.913	0.544			
Tumor size	(< 5 / ≥ 5)	163/134	3.339	1.605–6.946	0.001	1.366	0.567–3.291	0.487
Differentiation	(well & mod/poor)	184/113	2.335	1.203–4.530	0.012	1.264	0.616–2.594	0.523
Pathological stage	(1,2 / 3)	224/73	7.720	3.796–15.699	< 0.001	4.178	1.714 -10.183	0.002
CEA	(< 5.0 / ≥ 5.0)	225/72	2.773	1.436–5.357	0.002	1.995	1.016–3.914	0.045
CRP	(≦ 0.5 / > 0.5)	248/49	1.566	0.712–3.444	0.264			
GNRI	(> 90.9 / < 90.9)	231/66	2.578	1.318–5.043	0.006	1.754	0.849–3.625	0.129
Complications	(absent/present)	202/95	1.706	0.872–3.339	0.119			
Intraope. blood loss	(< 50 / ≥ 50)	(163/134)	1.989	0.955 – 4.141	0.066			
Adjuvant chemo	(no/yes)	218/79	4.190	2.142–8.198	< 0.001	1.761	0.795–3.900	0.163

Abbreviations: HR: hazard ratio, CI: confidence interval, ASA-PS: American Society of Anesthesiologists physical status, BMI: body mass index, mod: moderately, poor: poorly, CEA: carcinoembryonic antigen, CRP: C-reactive protein, GNRI: geriatric nutritional risk index, Complications: postoperative complications, Intraope. blood loss; intraoperative blood loss, Adjuvant chemo: adjuvant chemotherapy.

### Postoperative OS analysis stratified by the GNRI

**Figure 1:**
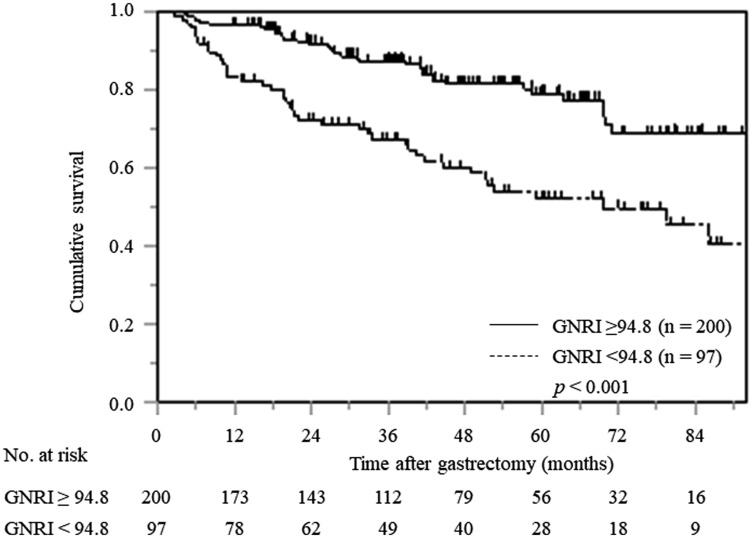
Kaplan–Meier curves of postoperative OS based on GNRI in all elderly gastric cancer patients.

Kaplan–Meier analysis and the log-rank test demonstrated that patients with a low GNRI had a significantly worse prognosis in terms of OS than those with a high GNRI (*p* < 0.001). The 5-year OS rates for patients with low and high GNRI values were 52.2% and 78.9%, respectively (Figure 1).

**Figure 2:**
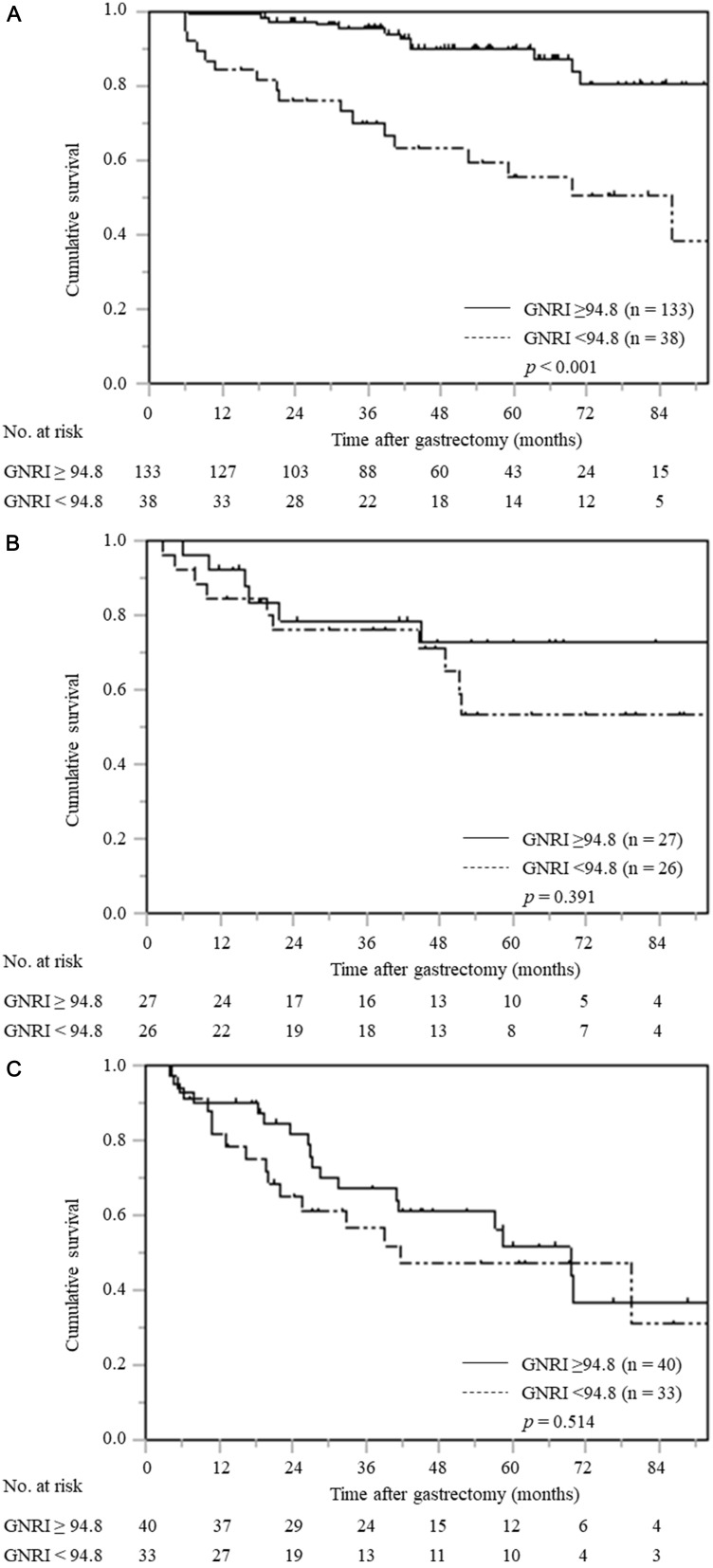
Overall survival curves based on GNRI according to pTNM stage. (**A**) pTNM stage I (*n* = 171), (**B**) pTNM stage II (*n* = 53), (**C**) pTNM stage III (*n* = 73).

In the stratified analysis according to pTNM stage, patients with low GNRI only in pTNM stage I had a significantly worse OS compared with patients with normal GNRI (*p* < 0.001); however, no significant differences were found among gastric cancer patients with pTNM stage II and III (Figure 2A–2C).

### Postoperative CSS analysis stratified by the GNRI

**Figure 3:**
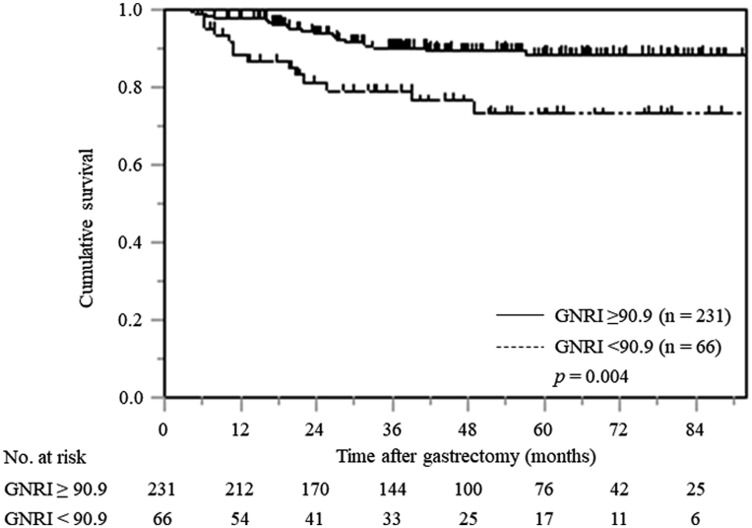
Kaplan–Meier curves of postoperative CSS based on GNRI in all gastric cancer patients.

Kaplan–Meier analysis and the log-rank test revealed a worse prognosis in terms of CSS in patients with a low GNRI than those with a high GNRI (*p* = 0.004). The 5-year CSS rates for patients with low and high GNRI values were 73.2% and 88.4%, respectively (Figure 3).

**Figure 4:**
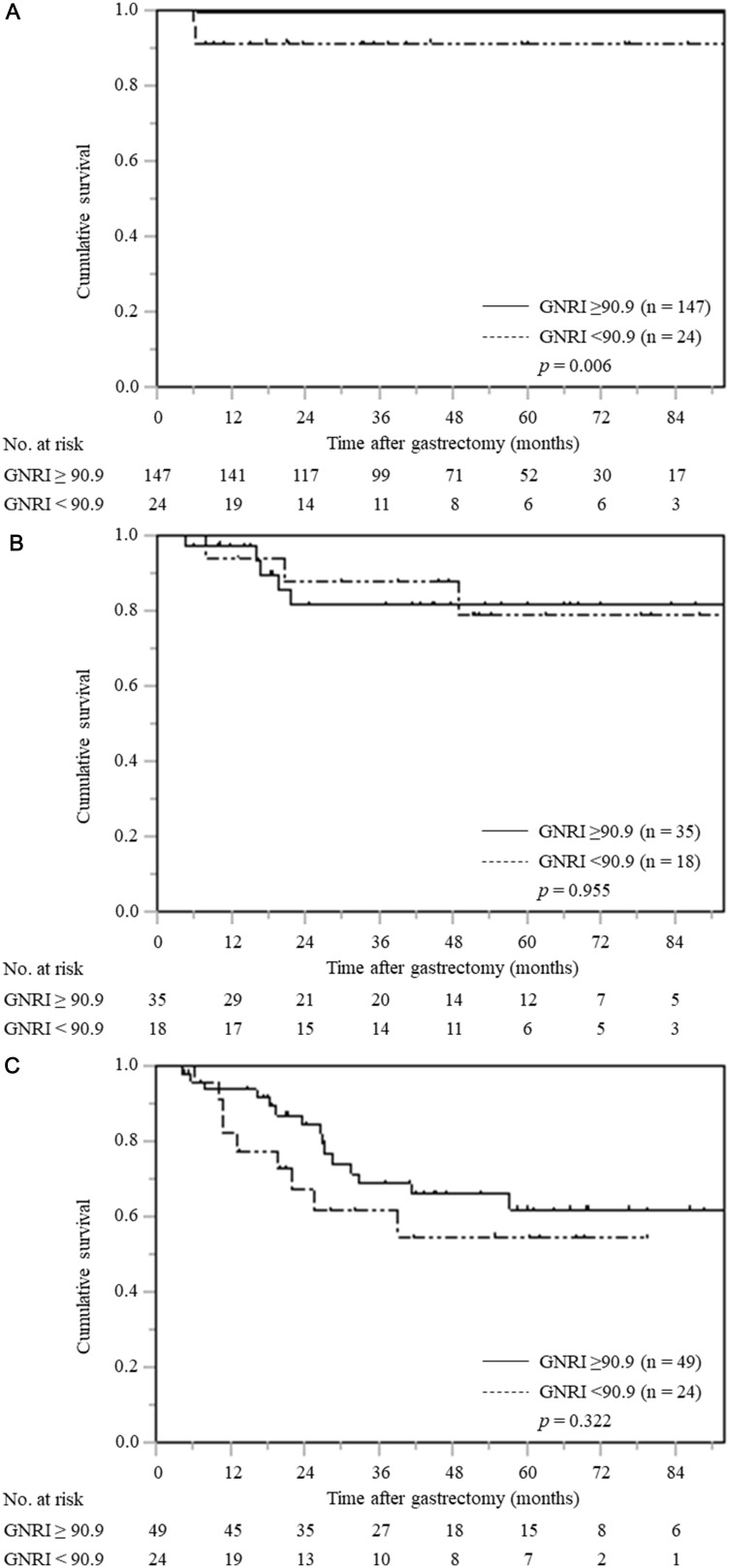
Cancer-specific survival curves based on GNRI according to pTNM stage. (**A**) pTNM stage I (*n* = 171), (**B**) pTNM stage II (*n* = 53), (**C**) pTNM stage III (*n* = 73).

The stratified analysis according to pTNM stage showed that patients with low GNRI had significantly worse CSS than patients with normal GNRI only in pTNM stage I (*p* = 0.006); however, there were no significant differences among gastric cancer patients with pTNM stage II and III (Figure 4A–4C).

## DISCUSSION

Malnutrition is a very common problem among the elderly. It is caused by age-related physical, psychological, or physiological changes [11], leading to diminished quality of life, performance status, immune function, and resistance to infections [12, 13]. Furthermore, malnutrition is particularly common in patients with gastrointestinal cancer because the disease mechanically inhibits the oral intake of food and/or directly impairs digestion either through local or systemic effects.

Several nutritional assessment tools have been developed to predict the prognosis of cancer patients, including the Glasgow Prognostic Score (GPS), the Controlling Nutritional Status (CONUT) score, and the Prognostic Nutritional Index (PNI) [14–16]. However, the clinical application of these tools in elderly cancer patients is limited because of the lack of consensus regarding their usefulness. The GNRI was originally developed as an objective and simple nutritional screening tool to evaluate the nutrition-related risks of hospitalized elderly patients. Patients were divided into four groups based on the GNRI values: high risk (GNRI < 82), moderate risk (GNRI 82–92), low risk (GNRI 92–98), and normal level (GNRI > 98) [5]. However, in this study, the long-term outcome of gastric cancer patients was examined regardless of their hospitalization status in order to create a cutoff value using receiver operating characteristic (ROC) analysis. Patients have also been classified into the low- and high-GNRI groups, and an association between low GNRI and mortality in elderly patients with chronic obstructive pulmonary disease, chronic kidney disease, or chronic heart failure was recently reported [17–20]. However, the use of the GNRI for cancer patients is not fully standardized, and its availability is limited because of the small number of studies reported.

In other contexts, GNRI has been reported as a useful tool for screening nutritional status, which is commonly determined using albumin and body weight loss [5]. Albumin stabilizes cell growth and DNA replication, participates in a variety of biochemical changes, protects against tumorigenesis, and maintains sex hormone homeostasis. Generally, hypoalbuminemia is commonly recognized in elderly populations caused by poor dietary intake, protein loss, or catabolic metabolism; therefore, albumin can reflect the inflammation and immune status of cancer cells [21, 22]. Likewise, most elderly patients are malnourished because of poor dietary intake, protein loss, or catabolic metabolism. Malnutrition is a complex state that is often related to functional impairment, disability, poor health, and aging, resulting in protein and energy deficiency, energy collapse, and impaired immunity. BMI, which considers the body weight and height, is also commonly used to assess an individual’s nutritional status [23, 24]. Therefore, GNRI, which is based on the combination of albumin and BMI, is a more accurate predictor of nutritional status than either of these parameters alone. As expected, a low GNRI adversely affected OS after laparoscopic gastrectomy in gastric cancer patients aged 65 or older in this study. However, it did not affect CSS. Although the mechanisms by which GNRI affects only provisional OS and not CSS is difficult to clarify, the possible reasons could be the increase in oral intake disorders that tends to occur with aging results in hypoalbuminemia and malnutrition [25]; cancer promotes inflammation and impairs nutrient absorption by increasing the production of catabolic cytokines, which accelerate malnutrition [26, 27]; and aging itself is associated with a systemic chronic low-grade inflammation; hence, a vicious cycle of malnutrition and inflammation occurs in gastric cancer patients, especially in the elderly [28–30]. We, therefore, believe that the GNRI can accurately predict OS because it reflects the integrated nutritional status associated with cancer progression and other age-related catabolic reactions.

In the future, the potential cancer-specific predictability of other prognostic nutritional scores such as the GPS, CONUT, and PNI, which consider albumin and other immune-nutritional factors, should be validated. Recently, several studies have indicated that a systemic inflammatory response may be associated with poor outcome in patients with advanced cancer. In particular, the GPS, an inflammation-based prognostic score that includes CRP and albumin, is one of the most useful scoring systems for prognosticating patients with various advanced cancers [31, 32]. CONUT, which is calculated using albumin, total lymphocyte, and total cholesterol, comprehensively reflects the balance between host inflammatory and immune responses because cholesterol is used as a parameter of caloric storage and lymphocyte is used as an indicator of immune status [16, 33]. Additionally, PNI, which is based on lymphocyte and albumin, was originally reported as a nutritional assessment tool for predicting the risk of operative morbidity after gastrointestinal surgery. Currently, PNI can be considered a good indicator of prognosis and the immune and nutritional status of patients with cancer [34, 35].

This study has some limitations that should be considered when interpreting the results. First, this study has a small sample size, which accounts for the lack of statistical power, and thus, we were unable to obtain sufficient information on preoperative comorbidities. To draw a better conclusion, a multicenter, large-scale study is needed to establish the role of GNRI as a prognostic predictor in patients with gastric cancer. Second, there are no consensual cutoff values for GNRI in predicting OS. The universal cutoff value should be verified in prospective and well-designed randomized controlled trials before it is adopted in routine practice. Third, the definition of elderly population is not consistent among researchers. Finally, GNRI has not been compared with other nutritional assessment tools; therefore, future prospective randomized studies are warranted to investigate the significance of preoperative nutritional intervention for improving surgical outcome in gastric cancer patients.

This study evaluated the association between preoperative GNRI and long-term outcomes in elderly gastric cancer patients. The GNRI, which was originally developed as an objective and simple nutritional screening tool to evaluate the nutrition-related risk index in elderly hospitalized patients, has been reported to be associated with mortality in elderly patients with chronic obstructive pulmonary disease, chronic kidney disease, and/or chronic heart failure. However, this is one of the few studies that assessed the use of GNRI for elderly patients with gastric cancer. In conclusion, GNRI proved to be a promising candidate not only for assessing the nutritional status of hospitalized elderly patients but also for predicting OS in elderly gastric cancer patients after laparoscopic gastrectomy because it reflects the integrated nutritional status associated with cancer progression and age-related catabolic reactions. When deciding a treatment strategy for cancer patients, tumor- and host-related factors should be considered. Recently, GNRI has been reported as an accurate tool to assess the risk of patients with chronic obstructive pulmonary disease, with cardiovascular disease, undergoing hemodialysis, and with chronic renal failure. In Japan, which has an aging society, individualized treatment strategy for gastric cancer is indispensable because there are many deaths caused by other diseases. Recently, sarcopenia has been reported to affect the incidence of adverse events with chemotherapy and the continuation of treatment, leading to worse prognosis. Sarcopenia, the age-related loss of skeletal muscle mass and strength, was identified based on cross-sectional computed tomography images at the L3 level [36]. However, GNRI can be easily calculated from routine laboratory data and physical measurements. The clinical significance of GNRI, as an indicator of OS, will be increasingly important in the future.

## MATERIALS AND METHODS

### Patients

We conducted a retrospective analysis of elderly patients with gastric cancer who underwent curative laparoscopic (assisted) gastrectomy at our institute between January 2010 and December 2017. The inclusion criteria were age ≥ 65 years (according to the World Health Organization’s definition of elderly; https://www.who.int/), histologically verified gastric adenocarcinoma, no distant metastasis, Eastern Cooperative Oncology Group performance status ≤ 2, and curative gastrectomy. The exclusion criteria were remnant gastric cancer, T4b disease, emergency gastrectomy for bleeding or perforation, mortality related to surgery within 30 days, and coexisting hematological, inflammatory, or autoimmune disorders. Based on these inclusion and exclusion criteria, a total of 297 patients were enrolled in the study. Our study was approved by the Ethical Review Board of the Shimane University Faculty of Medicine, Shimane, Japan (authorization number: 4011).

### Outcome evaluation

The median follow-up time was 36.0 months (range: 2.8–96.5 months) in all patients after gastrectomy.

The observation period started on the date of gastrectomy and ended on the date of death, last follow-up, or withdrawal of consent. OS and CSS were assessed based on the cause of death as determined from manual or computerized records. OS was calculated using the period from the date of primary gastrectomy to the date of death from any cause or last follow-up. CSS was defined as the interval from the date of surgery to cancer-related death or withdrawal of consent.

The pathological stage was assigned in accordance with the TNM classification (7th edition) [1]. We categorized postoperative complications according to the Clavien–Dindo (CD) classification, and postoperative complications were defined as grade II or higher [37].

### GNRI calculation

Preoperative laboratory data, including the GNRI, and physical measurements were obtained in each patient within 7 days prior to surgery.

The GNRI was developed by combining two nutritional indicators: albumin and actual weight compared with ideal body weight. The GNRI formula is as follows: GNRI = [1.487 × serum albumin (g/L)]+[41.7 × actual/usual body weight) (kg)]. The formula for the ideal body weight is as follows: ideal body weight (kg) = 22 × square of height (m^2^) [5]. When the patient’s actual BW is greater than the ideal BW, the ratio of the actual BW to the ideal BW was 1.

**Figure 5:**
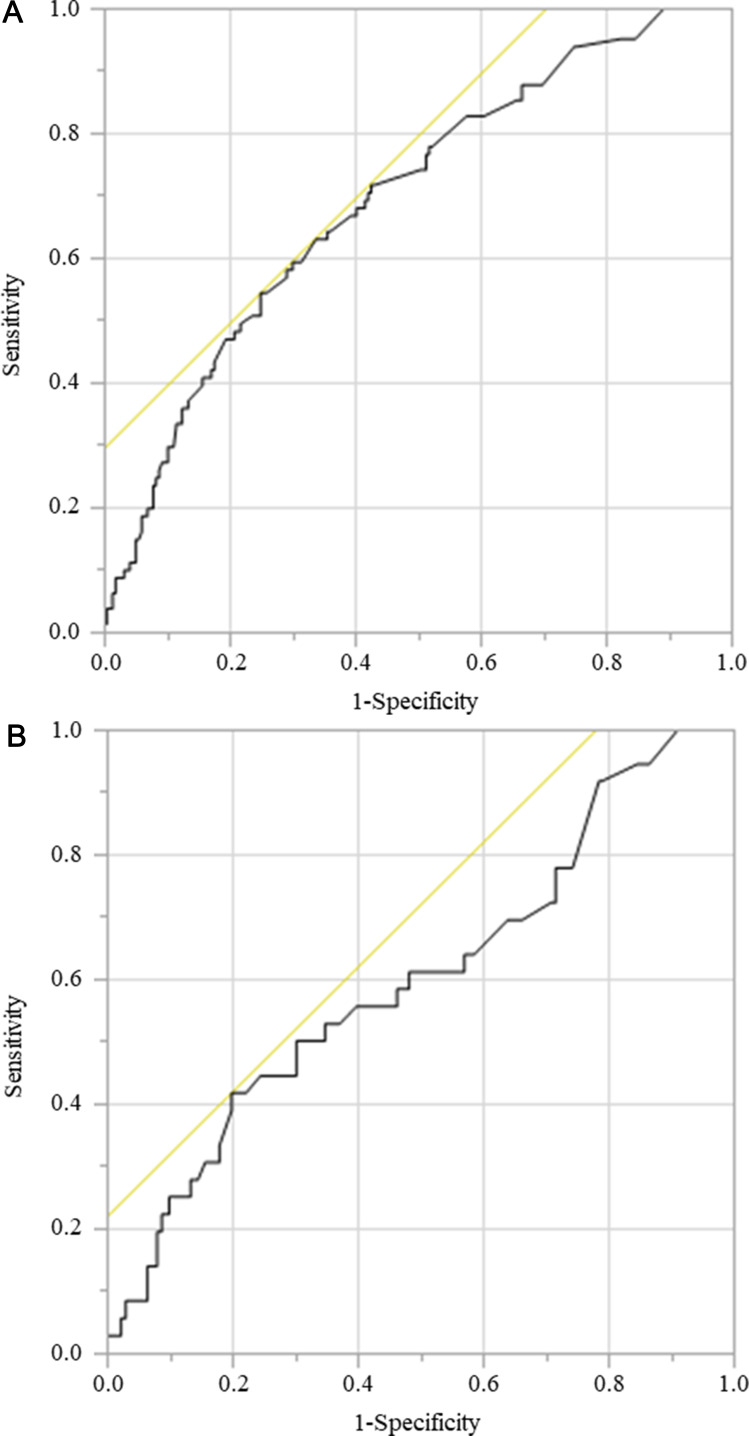
ROC for GNRI as a predictive factor for postoperative survival was plotted to verify the optimum cutoff value of GNRI. (**A**) overall survival, (**B**) cancer-specific survival.

A ROC curve was generated to verify the optimal cutoff value of preoperative GNRI for predicting OS and CSS. The ability of GNRI to predict OS and CSS was evaluated using the area under the ROC curve (AUC) estimation method. The GNRI ranged from 54.4 to 117.6, and the mean GNRI was 98.5. The median GNRI was 99.9. The optimal cutoff value for preoperative GNRI was set at 94.8 for OS (sensitivity, 54.3%; specificity, 75.0%; AUC = 0.694) and 90.9 for CSS (sensitivity, 41.7%; specificity, 80.1%; AUC = 0.599). Patients were categorized as having a low or high GNRI based on the cutoff values (Figure 5A, 5B).

### Statistical analysis

Comparisons were performed using the chi-square test or Student’s *t*-test for categorical variables and the Mann–Whitney *U* test for non-normally distributed continuous variables. The OS and CSS were calculated using the Kaplan–Meier method, and the differences between the survival curves were assessed by the log-rank test.

Univariate analyses were also performed to identify variables that were significantly associated with OS or CSS, and variables with a univariate *p* value < 0.05 were included in the multivariate analysis, for which a Cox proportional hazards model was used. JMP version 14 (SAS Institute, Cary, NC, USA) was used for the statistical analyses.

## Abbreviations

ASA-PS: American Society of Anesthesiologists physical status
AUC: area under the curve
BMI: body mass index
CD: Clavien–Dindo
CEA: carcinoembryonic antigen
CONUT: Controlling Nutritional Status
CRP: C-reactive protein
CSS: cancer-specific survival
GNRI: geriatric nutritional risk index
GPS: Glasgow Prognostic Score
HR: hazard ratio
OS: overall survival
PNI: Prognostic Nutritional Index
RBC: red blood cell
ROC: receiver operating characteristic
TNM: tumor–node–metastasis
